# Early-life circumstances and late life loneliness trajectories among Finnish older adults

**DOI:** 10.1186/s12877-024-04967-6

**Published:** 2024-05-24

**Authors:** Elisa Tiilikainen, Marja Aartsen, Siiri-Liisi Kraav

**Affiliations:** 1 Department of Social Sciences, University of Finland, Helsinki, Finland; 2 NOVA - Norwegian Social Research, Oslo Metropolitan University, Oslo, Norway

**Keywords:** Loneliness, Trajectories, Older adults, Aging, Early-life circumstances, Childhood adversities, Latent class analysis

## Abstract

**Background:**

Later life loneliness has become a significant public health concern worldwide. Research has focused on the prevalence, risk factors and consequences of loneliness in different age groups. This study aimed to advance the understanding of the impact of early-life circumstances on later life loneliness by examining the associations between adversities in childhood and youth and loneliness trajectories in Finnish older adults.

**Methods:**

The data were derived from the 10-year follow-up survey study Good Aging in the Lahti Region (*n* = 1552, mean age 64.89 years). The baseline study was conducted in 2002 with a regionally and locally stratified random sample of older persons living in the Lahti Region located in southern Finland. The follow-up surveys were carried out in 2005, 2008 and 2012. Loneliness was measured using a single question at the three follow-ups. Childhood conditions were retrospectively assessed at baseline with questions regarding the death of parents, household affection, relocation, and fear of a family member. Latent class growth analysis with time invariant covariates was used to identify loneliness trajectories and to examine the associations between loneliness trajectories and adverse circumstances in childhood and youth.

**Results:**

The results identified three distinct loneliness trajectories: low, moderate, and severe, including 36%, 50% and 14%, respectively, of the study population. The non-significant slopes of the three trajectories indicate that trajectories were stable during the seven years of follow-up. Being afraid of a family member, having a cold childhood, and death of a father or mother in childhood or youth significantly increased the odds of having a severe loneliness trajectory as compared to low loneliness trajectory. None of the early-life circumstances differentiated between severe and moderate levels of loneliness.

**Conclusions:**

The findings suggest that some adverse early-life circumstances increase the odds of an unfavorable loneliness trajectory in later life. The results highlight the need to recognize the role of diverse life-course adversities in loneliness research and interventions. The study also underscores the importance of identifying individuals who are at risk of long-term and severe loneliness and providing them with appropriate support to decrease and/or prevent the negative health consequences of loneliness in old age.

**Supplementary Information:**

The online version contains supplementary material available at 10.1186/s12877-024-04967-6.

## Introduction

Loneliness has been referred to as one of the new “geriatric giants”, leading to severe health consequences in old age [1]. Among the many negative outcomes are a lower level of cognitive functioning and faster cognitive decline [2, 3], cardiovascular diseases [4], cancer incidence [5], increased use of social and health services [6, 7], and mortality [8].

The prevalence of loneliness is known to vary with age and place. Several studies have shown that the highest prevalence of loneliness occurs in the youngest (< 30) and oldest (> 75) age groups [9, 10]; others find a linear decrease in loneliness with age worldwide [11]; others indicate that age differences in the prevalence of loneliness depend on the country or culture in which people live [9, 12, 13]. The factors consistently found to be associated with loneliness are partner status and widowhood, social network size, depression, self-perceived health, and social activities [14].

Despite a substantial amount of related research, only a few studies have grasped the temporal changes and/or stability of loneliness in old age [15, 16]. Moreover, very little is known about how different life-course factors shape the intensity and duration of loneliness later in life. Our study aims to fill these gaps by examining how trajectories of loneliness vary in older people and how adverse early-life circumstances are associated with different loneliness trajectories.

Based on existing conceptualizations, we understand loneliness as a negative and unpleasant emotional state expressing a mismatch between an individual’s desires or expectations toward social relationships and the perceived reality of one’s social life. This evaluation process is affected by past experiences and the experiences of others [17, 18]. Moreover, we recognize that there is strong variation in the ways people cope with loneliness and that people can go in and out of states of loneliness [17, 19].

Typologies, such as situational and chronic loneliness [15, 20] and trait and state loneliness [21], indicate that the duration of loneliness varies between stable and nonstable during the life course, leading to diverse health impacts. Empirical longitudinal studies, on the other hand, have shown three to five different loneliness trajectories in older adults, including forms of increasing, decreasing and stable loneliness and, in some cases, fluctuating loneliness [22–26].

Compared to studies among younger age groups [27, 28], only a few studies on older adults have established factors influencing different trajectories. In a five-year follow-up, Newall et al. [26] found that persistent loneliness was associated with living alone, being widowed or divorced, having poor health, and experiencing low perceptions of control. In a 20-year follow-up study, Solomon et al. [29] found that loneliness remained stable among veterans with antecedent combat stress reaction (CSR) but decreased among veterans without CSR. Persistent loneliness was also associated with higher levels of posttraumatic symptoms and lower levels of social support. Both studies highlight the importance of examining loneliness from a longitudinal perspective but focus only on adult or later life conditions and not on previous life circumstances.

## Early-life circumstances and loneliness

As early as the 1950s, at the dawn of loneliness research, Sullivan [30] suggested that loneliness in childhood foreshadows loneliness later in life [31]. Later, empirical evidence started to support this idea. Marangoni and Ickes [32] emphasized the relevance of childhood circumstances for later life loneliness and suggested that different subgroups of people exist and that for some people, feelings of loneliness persist throughout the life course since childhood.

Quantitative studies on childhood circumstances and loneliness have revealed various factors that are associated with loneliness in different age groups, such as poverty [33, 34], parents’ substance abuse [34, 35], sexual abuse [36], quality of relation with parents [37], and divorce or death of the parents [38]. Moreover, some qualitative studies have examined associations between trajectories of loneliness and early-life circumstances and found that severe and long-term loneliness are influenced by childhood events and experiences, such as being bullied in peer relations, the death of a parent and sexual abuse [35, 39].

Theoretically, associations between loneliness and early-life circumstances have been interpreted through John Bowlby’s [40] concept of early attachment, for example, suggesting that attachment failures in childhood and challenges in early attachments can lead to loneliness later in life [41]. Moreover, these associations can be explained through childhood influences on personality characteristics, such as self-esteem and self-efficacy [18, 42], and from the perspective of cumulative disadvantages [43], through which long-term hardship may impact psychological and social well-being in later life [34]. However, knowledge of adverse early-life circumstances and later life loneliness has been limited and particularly scarce from the perspective of long-term loneliness.

## Study aim and hypotheses

Adding to the existing understanding of life course influences on healthy aging [44], in this study, we examine associations between adverse early-life circumstances and seven-year loneliness trajectories in later life. In line with previous findings, we hypothesize that adverse circumstances in childhood and youth are associated with higher trajectories of loneliness (H1), irrespective of the type of event or experience (H2).

## Research design and methods

### Data

The data used in this study is derived from a longitudinal study in Finland: Good Aging in the Lahti Region (GOAL program, in Finnish: Ikihyvä Päijät-Häme) [45]. The baseline study was conducted in 2002 with a regionally and locally stratified random sample. The program approached 4,272 persons living in the Lahti Region located in southern Finland, and the response rate was 65.8%, leading to a baseline sample of 2814 persons. The participants were born between 1926 and 1930, 1936–1940, and 1946–1950, with ages of 52–56, 62–66 and 72–76 years, respectively, at baseline in 2002. The follow-up surveys were carried out in 2005, 2008 and 2012, and during the first follow-up in 2005, the sample was increased by 102 persons due to the inclusion of one new municipality in the study region. At baseline, the basic characteristics of the participants and retrospective information on circumstances in childhood and youth were assessed, while loneliness was introduced in the questionnaire in 2005 and was also part of the follow-ups of 2008 and 2012. The participation rate at the follow-ups ranged between 49% and 66% [46].

Included in the study sample are participants who participated at baseline (2002) and who had at least two follow-up observations of loneliness (*N* = 1829). In the latent class growth models, a further selection of people without missing observations on the covariates resulted in a final study sample of 1,552 people. Missing values on the loneliness variable were taken into account by means of the MLR estimator, which is robust to non-normality. Dropout during follow-up was associated with employment and current life situation: in the youngest cohort, the unemployed were more likely to drop out of the study, and those most likely to continue were people living with a partner. Overall, the older population in this study is socially and physically somewhat more active, more educated, and has better health and well-being than the total sample [46].

### Measurements

#### Loneliness

Loneliness was measured with a single question, “Do you feel lonely?” with five alternative answers: “never (1),” “seldom (2),” “occasionally (3),” “often (4)” and “all the time (5)”. The limitations and benefits of the single question are examined in the discussion section.

#### Predictors of loneliness trajectory

Guided by previous studies and availability in the dataset, we included characteristics of childhood and youth as predictors of loneliness trajectories in later life. As the survey did not include a validated measure of childhood adversities, we included a set of individual questions, including the death of the father, death of the mother, household level of affection, relocation in childhood, being afraid of a family member, and the total number of adverse early-life circumstances. All measures related to childhood and youth were based on retrospective information asked in 2002.

#### Early-life circumstances

Parental bereavement was measured with two separate questions inquiring about the year when the participant lost the mother and the father. Death of mother was recoded into (1) if the mother died before the age of 18, and into (0) if the mother did not die before the age of 18. A similar procedure was used for the death of the father.

Household level of affection reflects the way respondents describe their childhood home. They were asked to indicate to what extent each of the following characteristics described their childhood home: W*arm, caring*; *Inspiring, encouraging*; *Quarrelsome; Trusting, understanding; Strict; Open; Unfair; Happy* and *Indifferent, not interested* with five alternative answers (1) Describes well (2), Describes fairly well (3), Describes to some extent (4), Describes poorly, and (5) Doesn’t describe it at all. To reduce the number of factors and increase the reliability of household affection, we calculated a scale of cold childhood based on the sum score of four individual items: Quarrelsome, Strict, Unfair, Indifferent, not interested (Cronbach alpha = 0.72). Scale scores ranged from 5 to 20, with higher scores indicating higher affection.

Relocation in childhood was measured using the question, “When you were of school age [7–16 years], did you move to a different municipality so that your best friends changed?” followed by a specifying question, “How many times during school age?”. The latter variable was re-coded into three categories: (0) No moves; (1) One move, and (2) Two or more moves during school age.

Being afraid of a family member was also used as an indicator of adverse early-life circumstances. For this we used the question, “When you think back to your childhood and youth: Were you afraid of a family member?” with three answering categories: (1) Not at all; (2) Sometimes, and (3) Often.

The total number of adversities in early-life is a count variable, reflecting the total number of the following adversities: death of the mother before the age of 18, death of the father before the age of 18, at least one relocation in childhood, having had often financial hardships, having been at least sometimes afraid of a family member, having had a family member with alcohol-related problems at least sometimes, and two indicators of the level of family affection; that is belonging to the 10% of people who had the lowest scores on a warm childhood (based on five items: Warm, caring; Inspiring, encouraging; Trusting, understanding; Open; and Happy) and belonging to the 10% of people who had the coldest childhood household.

### Statistical analysis

The analysis consisted of several steps. After describing the basic characteristics of the study sample, we conducted Latent Class Growth Analyses (LCGA), which is a person-centered approach used to (1) identify distinctive loneliness trajectories, and (2) estimate the proportion of the study population following each trajectory. By including covariates (childhood characteristics and other demographic variables), we (3) related the probability of a certain trajectory to individual characteristics for each covariate by means of a multinomial regression [47]. The LCGA was conducted in a stepwise manner. First, we estimated an unconditional single latent class growth model, i.e., a model that includes only the loneliness observations at the subsequent waves and only one class or trajectory to derive the estimates for the growth parameters. Factor loadings of the intercept were fixed to be equal across time to ensure that the same concept is measured over time (factorial invariance).

Next, in accordance with other studies [47–49], we decided upon the optimal number of trajectories based on the Bayesian Information Criterion (BIC), the Bootstrapped LR difference test (b-LRT), an entropy summary statistic, the number of people assigned to each trajectory, and the meaningfulness of the trajectories. Extra trajectories were added to the model as long as the Bayesian Information Criterion (BIC) continued to decrease [49], and the b-LRT, which tests the − 2 log likelihood difference between a model with k classes and k − 1 classes, was significant.

The last indication for consideration about the number of classes is a high entropy value (near 1.0) and trajectories that contain at least 5% of the cases [48]. Entropy gives an indication of the classification accuracy of individuals into the trajectories [50]. The within-class variance of the growth parameters was fixed to zero, which makes sense since we assume that all individuals within a certain latent class have the same trajectory of loneliness [48].

Once the number of trajectories was defined, we added the early-life circumstances and control variables and regressed the latent class variable on the selected covariates to estimate the probability of a certain trajectory given the value of the covariates. Mplus version 8.4 [51] was used in the current study.

## Results

Descriptive statistics of all study variables can be found in Table 1. 53.4% of the study population was female. The mean age at the first follow-up (2005) for all participants was 64.89 years. 5.3% of participants had lost their mother and 13.1% their father before the age of 18. 29.2% had at least one relocation during childhood, 19.9% of participants had often encountered financial difficulties in their childhood homes, and 31.6% had been at least sometimes afraid of a family member. The mean number of adversities experienced in childhood was 1.48.

**Table 1:** Descriptive statistics of the study population

Variable	N (Valid data)	%	M (SD)	Missing data (%)
Age	1552		64.89	0.00
Female	828	53.40		0.00
Mother died before the age of 18	83	5.30		0.00
Father died before the age of 18	204	13.10		0.00
Relocation in childhood				0.00
No moves	1099	70.80		
One move	213	13.70		
Two or move moves	240	15.50		
Being afraid of a family member				0.00
Not at all	1062	68.40		
Sometimes	424	27.30		
Often	66	4.30		
Cold childhood environment	1552		15.71 (2.99)	0.00
Number of childhood adversities (0–8)	1552		1.48 (1.38)	0.00
Feels lonely in 2005 (0–5)	1536		1.81 (0.80)	1.00
Feels lonely in 2008 (0–5)	1495		1.87 (0.79)	3.70
Feels lonely in 2012 (0–5)	1271		1.79 (0.81)	18.10

The correlations of the study variables are presented in Table 2. The bivariate correlations between the three loneliness measures were rather high, suggesting substantial stability over time. Death of the father or mother before the age of 18 was not significantly associated with later life loneliness. Relocation at T1 and T2 was associated with higher levels of loneliness. Being afraid of a family member was associated with higher levels of loneliness at all three time points, and the negative correlation between cold childhood environment and loneliness indicates that a higher score on the cold childhood scale (indicative of higher affection) was inversely associated with loneliness. The number of adverse childhood events was positively associated with the three loneliness observations, indicating that a higher number of events related to higher levels of loneliness. Associations between age and loneliness did not reach the level of significance.

**Table 2:** Bivariate Correlations between the study variables

		1	2	3	4	5	6	7	8	9	10
1	Loneliness T1	1.00									
2	Loneliness T2	**0.60**	1.00								
3	Loneliness T3	**0.55**	**0.56**	1.00							
4	Mother died before the age of 18	0.05	0.02	0.00	1.00						
5	Father died before the age of 18	0.02	0.03	0.02	0.02	1.00					
6	Relocation in childhood	**0.05**	**0.07**	-0.01	**0.06**	-0.03	1.00				
7	Being afraid of a family member	**0.11**	**0.10**	**0.09**	-0.03	-0.01	0.01	1.00			
8	Cold childhood environment	**-0.13**	**-0.13**	**-0.14**	0.05	0.04	**-0.05**	**-0.53**	1.00		
9	Number of childhood adversities	**0.10**	**0.11**	**0.07**	**0.16**	**0.26**	**0.37**	**0.65**	**0.56**	1.00	
10	Age	0.03	0.03	0.11	0.07	0.10	0.10	-0.07	0.11	0.00	1.00

*Note* Significant (*p* < 0.05) associations in bold

In the final models, we used 500 initial stage random sets of starting values and 40 final stage optimizations, and we used 20 starting iterations to avoid ending the estimation in a local optimum and found that the best loglikelihood value was repeated. The best solution for the Latent Class Growth Models was a model with three trajectories. Compared to a model with two trajectories, the three-class model had a lower BIC, the b-LRT was significant and the classification quality (entropy) was better. While also a four-class model has a significant b-LRT, all other indices were worse and one class was only observed in less than 0,01% of the study sample (Table 3).

**Table 3:** Fit indices and trajectory class proportions of the latent class growth analyses

	Fit statistics					Proportions for the latent classes			
	AIC	BIC	AdjBIC	Entropy	b-LRT (*p*)	1	2	3	4
2	8799.89	8880.10	8832.44	0.70	0.00	0.47	0.53		
3	8510.23	8643.91	8564.49	0.72	0.00	0.36	0.50	0.14	
4	8530.23	8717.39	8606.20	0.78	0.00	0.36	0.00	0.50	0.14

*Note* b-LRT = Bootstrapped Likelihood Ratio Test for k-1 versus k classes

The proportions for the latent classes (Table 3) indicate that 36% (*n* = 556), 50% (*n* = 772) and 14% (*n* = 214) of the respondents could be assigned to class 1,2 and 3 respectively. Class one is the class with the lowest loneliness trajectory, class two refers to the moderate loneliness trajectory and class three refers to the severe loneliness trajectory.

The conditional latent class growth analysis indicated that, compared to people with a severe loneliness trajectory, people in the lowest loneliness trajectory scored low on all adverse early-life circumstances; they less often lost their mother (OR = 0.54, *p* = 0.04) or father (OR = 0.59, *p* = 0.02) before the age of 18 years, had less often been afraid of a family member in their youth (OR = 0.59, *p* < 0.001), and they less often indicated that household level of affection could be characterized as cold (OR = 1.16, *p* = 0.01). The total number of adversities, however, did not further raise the odds of having a severe loneliness trajectory beyond the impact of the single adversities in childhood and youth. None of the examined early-life circumstances differentiated a severe loneliness trajectory from the moderate trajectory (Table 4).

**Table 4:** Results from conditional latent class growth analyses (*N* = 1552)

	Loneliness class comparisons			
	Severe versus Low		Severe versus Moderate	
*Predictors*	OR	*p*	OR	*p*
Age	**0.97**	**0.02**	0.98	0.07
Mother died before age 18(Yes = 1; No = 0)	**0.54**	**0.04**	0.83	0.60
Father died before age 18 (Yes = 1; No = 0)	**0.59**	**0.02**	0.86	0.60
Moved to different municipality (0 = No; 1 = One time; 2 = Two or more times)	0.85	0.26	1.10	0.56
Being afraid of a family member (1 = No;2 = Sometimes;3 = Often)	**0.59**	**< 0.01**	0.88	0.54
Cold childhood (Higher scores indicate less cold childhood)	**1.16**	**< 0.01**	1.04	0.30
Number of childhood adversities	1.12	0.39	0.87	0.24

*Note* In bold significant OR’s

## Discussion

In this study, we examined trajectories of later life loneliness and their associations with early life circumstances, including negative or adverse experiences in childhood and youth. In line with previous research, the findings on loneliness trajectories showed that there was substantial stability in the intensity and duration of loneliness, and that most older adults experienced loneliness rarely or occasionally over time. However, almost one in seven older adults experienced severe loneliness during the three follow-up measurements, suggesting chronic loneliness [52]. The findings also indicated some adverse experiences in childhood and youth that were associated with long-term loneliness (both moderate and severe). Being afraid of a family member, having a cold childhood household and death of parent in childhood or youth significantly increased the odds of having a high loneliness trajectory compared to people who were not lonely.

There may be several reasons why a child could be afraid of a family member or experience coldness in the childhood environment, for example, different forms of abuse, authoritative parenting styles, or parents’ mental health problems. Based on existing research in the Finnish context, it is known that older generations have often experienced adversities in childhood families due to the impact of Finnish wars between 1939 and 1945. During these wars, most Finnish fathers were forced to go to the war front and, if not deceased, many came back home with trauma that might have led to problems with alcohol and mental health [39, 53, 54]. The older adults who participated in this study were born before, during, or soon after the war. Moreover, they had lived their childhood and youth during a time when children’s wellbeing and rights were not yet systematically recognized [55], and therefore may not have received adequate care and support in family disruptions.

Contrary to our expectations, we did not find an association between moving to a new municipality when comparing the severe loneliness trajectory to the low loneliness trajectory. One reason could be that the question did not fully capture the social aspects of relocation in childhood we had in mind, including the negative influence of the loss of friends. It may also indicate that, compared to later life, a young person is more flexible to changes in the living environment and that peer relations have a shorter history and can therefore be compensated through new relationships more easily. Interestingly, our finding regarding the death of father or mother in childhood or youth differed from previous research in Finland, where no association was found between parental bereavement and later life loneliness [56]. A possible explanation for this is the difference in the study population (the participants were born at a different time) and the previously discussed societal factors, including the impacts of war, which may have been different for younger cohorts in the older population. The impacts of parental bereavement on loneliness may also differ depending on the cause and circumstances following death [39].

We did not find that the total number of adversities increased the odds of a severe loneliness trajectory beyond the impact of a single adversity in early life. This differs from previous research underlining the co-occurring nature of childhood adversities [57] and their cumulative negative effect on physical health in older age [58, 59]. The predictive variables we used – death of parents, moving to a new municipality, being afraid of a family member, and a cold childhood – are all factors that by their nature are likely to be disruptive to social well-being in childhood and youth. Therefore, it is possible that even one of these experiences during early life is sufficient to increase the odds of developing high loneliness in older age. In the bivariate correlations the number of childhood events has a high correlation with a cold childhood (*r* = 0.56) and being afraid for a family member (*r* = 0.65). Since these two variables entered the equation first, the effect of the number of events may already be covered by these two variables. An alternative interpretation is that negative and adverse experiences in early life have a life-long impact, as they compromise the development of social and emotional skills needed to build satisfying relationships in later life [60].

Overall, the main findings of this study are in line with previous research, which found connections between loneliness and different adverse early life circumstances, such as violent environments, family members’ mental illnesses, and substance abuse [34, 61–64]. Broadening from our focus on loneliness, there is increasing evidence of an association between adverse childhood circumstances and mental health conditions, such as depression and anxiety [63, 65–68]. Both factors have critical impacts on the health and well-being of older adults. The connection between depression and loneliness is known to be particularly strong in later life [69, 70], but there are differences in the interpretation of the relationship and its direction [71–73]. In future research, it would be important to examine the relationship between depression and long-term loneliness in more detail from the perspective of early-life circumstances.

### Limitations and strengths

This study has some limitations that are important to consider when interpreting the results. The measurement of loneliness was based on a single question, “Do you feel lonely?”. This approach might not only underestimate nuances in the severity of loneliness but also limit variation in loneliness compared to multi-item scales and, therefore, statistical power. A more nuanced scale and more detailed estimation of the level of loneliness could better detect the interconnections of different childhood circumstances. The low association between loneliness and the total number of adverse early-life circumstances may also be a sign of weakness in the measurements and their interpretation as childhood adversities.

In this study, we were unable to use validated measures, such as the Prevalence of Childhood Exposure to Abuse and Household Dysfunction Scale (ACE; [58]) or the Childhood Trauma Questionnaire (CTQ; [74]), which have been commonly used when investigating childhood adversities. However, the set of questions we used was somewhat comparable to the items used in both scales (e.g., being afraid of a family member and household affection). Moreover, we were able to combine two approaches often used in studies on childhood adversities: examining different types of maltreatment in early life [75–77] and measuring the cumulative effects of negative childhood experiences and conditions [58, 78].

From the perspective of limitations, it is important to note that, as the follow-up period was only seven years and loneliness was measured only three times, the chronicity of loneliness in the group with higher loneliness trajectories is uncertain, as we do not know how long they had experienced loneliness before the first measurement. Moreover, as mentioned, a single question on loneliness may detect differences and changes in loneliness more poorly than multiple question scales. However, previous studies have also shown that single loneliness questions classifying respondents as lonely when they express feeling “often” or “always” are highly similar to single questions and aggregated scales [79], and that the single-item scale is suitable for assessing change and does not have major shortcomings when compared to longer scales [80].

In our study, information on childhood circumstances was gathered retrospectively and subjectively when respondents were aged 52 years and older. This is common in research on later life influences of childhood adversities, but it is important to note that such retrospective accounts may be compromised by poor recall [81]. Current loneliness may also affect the recall of childhood events, leading respondents to emphasize their negative experiences [34]. In this study, questions related to childhood circumstances were asked at baseline and not at the same time as questions about loneliness, which were assessed only in the follow-up surveys. Therefore, it may be assumed that reminiscing about childhood conditions did not impact how questions about loneliness were answered.

Despite the limitations of validated and multidimensional measurements, a key strength of this study is the inclusion of diverse retrospective questions regarding childhood conditions and experiences in the baseline. Early life circumstances and life course influences are still often ignored in gerontological research, despite the growing understanding of the “long arm” of childhood conditions on the health and well-being of older adults. Another strength of the study is the acknowledgement of different temporalities of loneliness and the inclusion of loneliness trajectories in the analysis, as later life loneliness is often examined with cross-sectional data focusing on loneliness during a single time point. In future research, more nuanced research strategies are needed to identify individuals who are at risk of long-term and severe loneliness and are exposed to the consequences of its chronicity [82].

### Conclusions and implications

The findings of this study show a connection between adverse early-life circumstances and long-term loneliness in later life, underlining the role of critical life course factors in the health and wellbeing of older adults. With respect to practical implications, the findings call for both preventive and corrective measures when aiming to reduce loneliness and prevent the negative health consequences of loneliness in older age. As for preventive measures across generations, focus is needed on ensuring well-being in childhood and youth and the provision of adequate support for families faced with diverse adversities. As for corrective measures, it is important that loneliness interventions are targeted and tailored for older adults experiencing long-term and severe loneliness, and that within these interventions, older adults are provided with the possibility to discuss and address past life events and experiences. For this, interventions implementing narrative and/or life-course-oriented practices may be especially beneficial.

### Electronic supplementary material

Below is the link to the electronic supplementary material.

Supplementary Material 1

## Data Availability

Access to the data is maintained by the Good Ageing in Lahti (GOAL, in Finnish Ikihyvä Päijät-Häme) research team. Requests to access these datasets should be directed to Professor Mikael Fogelholm, mikael.fogelholm@helsinki.fi.
