# Supplementary material for: Early-life circumstances and late life loneliness trajectories among Finnish older adults
Source: BMC Geriatr. 2024 May 24;24:459. doi: 10.1186/s12877-024-04967-6 (PMC11127366; doi:10.1186/s12877-024-04967-6)
Supplement: Supplementary file 1 — Supplementary Material 1 [file 12877_2024_4967_MOESM1_ESM.docx]

Appendix

Table 4b Results from conditional latent class growth analyses (N = 1552), two class solution

|  | Class comparisons | | | |
| --- | --- | --- | --- | --- |
|  | High versus Low | |  | |
| *Predictors* | OR | p |  |  |
| Age | 0.99 | 0.14 |  |  |
| Mother died before age 18(Yes=1; No=0) | 0.65 | 0.06 |  |  |
| Father died before age 18 (Yes=1; No=0) | **0.62** | **<0.01** |  |  |
| Moved to different municipality (0=No; 1=One time; 2= Two or more times) | **0.78** | **<0.01** |  |  |
| Being afraid of a family member (1=No;2=Sometimes;3=Often) | **0.66** | **<0.01** |  |  |
| Cold childhood (Higher scores indicate less cold childhood) | **1.12** | **<0.01** |  |  |
| Number of childhood adversities | **1.24** | **0.02** |  |  |
